# Emerging concepts in ventilation-induced lung injury

**DOI:** 10.12688/f1000research.20576.1

**Published:** 2020-03-31

**Authors:** Purnema Madahar, Jeremy R. Beitler

**Affiliations:** 1 Center for Acute Respiratory Failure, Columbia University College of Physicians and Surgeons, New York City, NY, USA; 2 Division of Pulmonary, Allergy, and Critical Care Medicine, Columbia University College of Physicians and Surgeons, New York City, NY, USA; 3 Department of Medicine, New York-Presbyterian Hospital, New York City, NY, USA

**Keywords:** ventilator-induced lung injury, acute respiratory distress syndrome, acute lung injury, respiratory mechanics, mechanical ventilation

## Abstract

Ventilation-induced lung injury results from mechanical stress and strain that occur during tidal ventilation in the susceptible lung. Classical descriptions of ventilation-induced lung injury have focused on harm from positive pressure ventilation. However, injurious forces also can be generated by patient effort and patient–ventilator interactions. While the role of global mechanics has long been recognized, regional mechanical heterogeneity within the lungs also appears to be an important factor propagating clinically significant lung injury. The resulting clinical phenotype includes worsening lung injury and a systemic inflammatory response that drives extrapulmonary organ failures. Bedside recognition of ventilation-induced lung injury requires a high degree of clinical acuity given its indistinct presentation and lack of definitive diagnostics. Yet the clinical importance of ventilation-induced lung injury is clear. Preventing such biophysical injury remains the most effective management strategy to decrease morbidity and mortality in patients with acute respiratory distress syndrome and likely benefits others at risk.

## Introduction

Acute respiratory failure often requires mechanical ventilation as a potentially life-saving intervention. However, the development of ventilation-induced lung injury (VILI) is a potentially life-threatening complication. The clinical importance of VILI is clear: limiting tidal volume has been shown definitively in two multicenter randomized trials to improve survival from acute respiratory distress syndrome (ARDS) ^1, 2^. Since then, lower tidal volumes have become standard of care for patients with ARDS ^3, 4^. Moreover, lower tidal volumes are used increasingly in patients without ARDS ^5^, and clinical data suggest benefit in patients at risk of VILI regardless of whether all criteria for ARDS are met ^6– 8^.

Despite current standard-of-care low tidal volume ventilation, some patients still may experience VILI ^9– 11^. Limited bedside diagnostic tools impede reliable diagnosis of VILI, and consensus is lacking regarding how best to individualize patient care for prevention and treatment.

The classic schema of VILI describes four central mechanisms: barotrauma, volutrauma, atelectrauma, and biotrauma ^12^. Recent preclinical studies and clinical trials have enhanced and refined our understanding of pathophysiology and may help prioritize when interventions offer competing effects on different mechanistic pathways.

This article will provide an updated view of VILI mechanisms, revisit strategies for bedside detection, and provide recommendations for personalized ventilator management to reduce the incidence of VILI guided by available evidence.

## Refining the classic schema of VILI mechanisms

### Barotrauma

The risk of positive pressure ventilation causing gross barotrauma, pneumothorax, pneumomediastinum, or subcutaneous emphysema, for example, has been recognized for centuries ^13, 14^. However, most VILI occurs without clinically overt barotrauma. In the classic ARDS Network trial that demonstrated improved survival targeting tidal volumes of 6 compared to 12 mL/kg predicted body weight (PBW) ^2^, there was no appreciable difference in gross barotrauma (10% versus 11%). For decades preceding ARDSNet, a more occult form of high-pressure injury also termed barotrauma has been recognized. In a classic experiment from Webb and Tierney ^15^, anesthetized, tracheotomized rats were ventilated with inspiratory pressures of 14, 30, or 45 cmH _2_O for one hour and compared to a control group that did not undergo positive pressure ventilation. The control and low-pressure (14 cmH _2_O) groups had no evidence of lung injury, whereas those targeting 30 or 45 cmH _2_O had increasingly severe histologic injury. Interstitial edema was evident at 30 cmH _2_O and both interstitial and alveolar edema at 45 cmH _2_O. Injury manifested during the experiment as hypoxemia, decreased respiratory compliance, and, in the highest-pressure group, death. Extrapolating these findings to the clinical setting, not only are alveolar edema, hypoxemia, and decreased respiratory compliance hallmarks of ARDS ^16, 17^ but they can also result directly from VILI. Thus, distinguishing between occult VILI, which may be preventable, and the sequelae of ARDS is exceedingly difficult.

### Volutrauma

The concept of volutrauma, as a potentially distinct form of injury from barotrauma, gained popularity following the report of a classic experiment by Dreyfuss and colleagues ^18^. In this study, rats were ventilated with one of three strategies targeting combinations of tidal volume and airway pressure: high-volume/high-pressure, high-volume/low-pressure, and low-volume/high-pressure. The high-volume/low-pressure strategy was achieved with negative pressure ventilation via an iron lung. The low-volume/high-pressure strategy was achieved by placing rubber bands around the thoracoabdominal region to restrict chest wall movement. The study found either strategy with high tidal volumes produced substantial lung injury, demonstrated by extravascular lung water, protein leak, and alveolar cell injury on electron microscopy. By contrast, the low-volume/high-pressure strategy experienced considerably less lung injury by all measures. The authors correctly concluded that “an increase in airway pressure without concomitant increase in lung volume does not produce pulmonary edema”. However, this finding has been misinterpreted by many as volutrauma being something different from, and more important than, barotrauma.

### Reconciling barotrauma and volutrauma

Barotrauma and volutrauma arguably describe related aspects of the same phenomenon and can be thought of as mechanical stress and strain, respectively ^19, 20^. For a deformation of lung shape (strain) to occur, a pressure must be applied (stress). Larger deformations may be injurious (volutrauma) and are generated by higher pressures (barotrauma).

Confusion often arises by misinterpreting airway pressure, which reflects respiratory system mechanics and does not differentiate lung from chest wall contribution ^21, 22^. Airway pressure is not a reliable indicator of lung parenchymal stress. The pertinent distending pressure of the lungs is the *transpulmonary pressure* (lung stress), the difference in pressure inside versus outside the lungs, equal to the airway minus pleural pressure ^23^. During a breath hold, when airflow and thus flow-resistive pressure are zero, transpulmonary pressure reflects only the elastic recoil of the lungs, often referred to as lung parenchymal stress ^24^. In the Dreyfuss experiment ^18^, the high-volume/low-pressure strategy was achieved via negative pressure ventilation, with an iron lung. Thus, while airway pressure was zero (atmospheric) at both end-expiration and end-inspiration, pleural pressure was more negative and transpulmonary pressure was greater at end-inspiration. Thus, the Dreyfuss high-volume/low-airway-pressure strategy is actually a high-volume/high- *transpulmonary*-pressure strategy. Changes in transpulmonary pressure (but not airway pressure) are concordant: larger changes in volume are accompanied by larger changes in transpulmonary pressure.

### Atelectrauma

**Figure 1.**
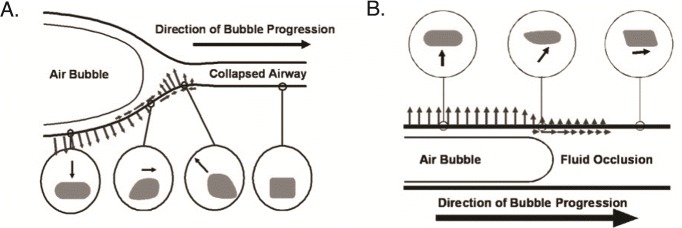
Local stress and strain of epithelial cells generated during recruitment of small airways. ( **A**) Air bubble propagation down the atelectatic airway generates a dynamic wave of stress and strain at the interface of the air bubble and collapsed airway. As the air bubble approaches, the epithelial cell is pulled inward toward the bubble. As the air bubble passes, the cell is pushed outward. ( **B**) The air bubble similarly generates stress and strain of epithelial cells during propagation along flooded airway. This figure was re-used from Ghadiali SN and Gaver DP, Biomechanics of liquid–epithelium interactions in pulmonary airways. *Respir Physiol Neurobiol* doi:10.1016/j.resp.2008.04.008 with permission ^28^.

Atelectrauma refers to injury resulting from the cyclic opening and closing of recruitable lung units (small airways extending to alveolar ducts and alveoli) during tidal ventilation ^25– 27^. In small airways, cyclic opening and closure with each breath generates injurious stress/strain along the airway epithelium as the opening airway takes an “unzippering-like” shape with air bolus propagation ( Figure 1) ^28, 29^. Similar locally high stress/strain may occur from cyclic recruitment/collapse of unstable alveoli that are predisposed to atelectasis because of surfactant inactivation ^30– 32^. Collapse is likeliest to occur at low end-expiratory lung volumes, when airway and transpulmonary pressures are comparatively lower. In preclinical models, the application of positive end-expiratory pressure (PEEP) to mitigate collapse and maintain higher end-expiratory lung volumes appears to mitigate lung injury ^25, 26, 32^.

However, whether atelectrauma leads to clinically significant lung injury in the modern era is unsettled. Most patients at risk of lung injury are managed with at least modest levels of PEEP in current practice ^33^. Clinical trials evaluating more aggressive PEEP strategies have not found a compelling benefit to higher PEEP, when either applied empirically ^34, 35^ or driven by respiratory mechanics ^36, 37^.

### Alveolar interdependence

**Figure 2.**
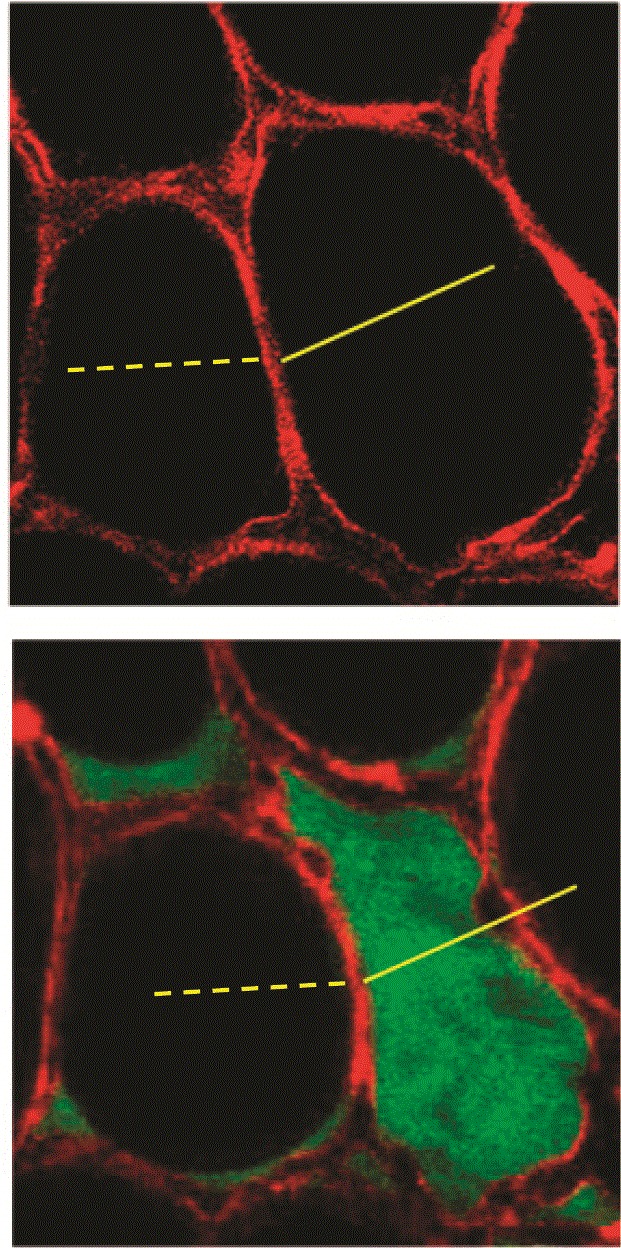
Spatial heterogeneity increases stress and strain due to alveolar interdependence. In the isolated perfused rat lung, confocal microscopy with optical sections 2 µm thick permits direct visualization of alveoli. *Top*: adjacent alveoli share a common septum and are mechanically interdependent. In normal conformation, strain is minimized across neighboring air-filled alveoli. *Bottom*: in a single-alveolus model of pulmonary edema, the effects on local strain distribution of heterogeneous parenchymal consolidation and flooding can be appreciated. The liquid-filled alveolus shrinks owing to micromechanical effects of meniscus formation. As a result, the adjacent air-filled alveolus bulges and overdistends. This figure was reprinted with permission of the American Thoracic Society. Copyright © 2020 American Thoracic Society. Cite: Perlman CE, Lederer DJ, Bhattacharya J. 2011. Micromechanics of alveolar edema. Am J Respir Cell Mol Biol, 44(1), 34–9. The American Journal of Respiratory Cell and Molecular Biology is an official journal of the American Thoracic Society ^40^.

Neighboring alveoli are mechanically interdependent and share an interalveolar septum. In the classic theoretical model proposed by Mead and colleagues ^23^, deformation of one alveolus, such as from collapse or being liquid-filled, necessarily causes deformation of the adjacent alveolus, generating high regional tensile forces. This theoretical model has been supported by several studies ^38– 40^. For example, Perlman and colleagues ^40^ used confocal microscopy to visualize neighboring alveoli and then microinfused one alveolus with albumin solution while leaving the others aerated. The liquid-filled alveolus shrunk, taking on the curvature of the meniscus formed at the mouth of the air–liquid interface. This shrinkage of the liquid-filled alveolus caused the interalveolar septum to stretch, bulging into the liquid-filled alveolus and leaving neighboring aerated alveoli deformed and over-expanded ( Figure 2). During lung inflation, the adjacent air-filled alveoli became more overdistended and deformed, predisposing to mechanical failure.

Clinical studies have observed greater inflammation in regions of radiographic heterogeneity ^41^, thought to be corollary findings to these microscopic observations. This form of VILI, resulting from heterogeneous stress/strain distribution both within individual alveoli and across neighboring alveoli, is described by several synonymous terms in the literature: stress raisers, stress concentration, and lung inhomogeneity, among others ^42, 43^. The concept, however, again speaks to deformation-induced lung injury. Much like high tidal volumes cause uniformly high stress and strain, mechanical heterogeneity from aerated alveoli adjacent to collapsed or liquid-filled alveoli leads to locally high stress and strain that gets magnified during tidal expansion ^41, 42, 44^.

### Patient self-inflicted lung injury

Re-thinking pressure changes during ventilation in terms of transpulmonary pressures makes it clear that biophysical lung injury may occur in the at-risk patient regardless of whether the ventilator or the patient is generating the pressure ^45^. This has led some experts to advocate adopting the term *ventilation*-induced lung injury or patient self-inflicted lung injury (PSILI) ^46– 48^. Regardless of terminology, the same basic principles of stress and strain, barotrauma and volutrauma, apply. Two important insights follow when considering patient respiratory effort. One, low airway pressures can be falsely reassuring if forceful inspiratory efforts are made in the mechanically ventilated, spontaneously breathing patient at risk of VILI ^49, 50^. Two, biophysical injury may occur even in the absence of positive pressure ventilation ^45^.

## Biotrauma: the final common pathway of VILI

Biotrauma refers to the biological response to mechanical injury. Regardless of the mechanism of VILI, mechanical cellular injury generates a regional and systemic inflammatory response that further propagates injury ^51– 54^. Release of damage-associated molecular patterns (DAMPs) in response to mechanical injury promotes the recruitment of immune cells that secrete pro-inflammatory cytokines ^55^. Paired with overstretch-induced activation of alveolar epithelial and vascular endothelial cell signaling cascades and dysregulation of the neuroinflammatory reflex ^56, 57^, a robust systemic inflammatory response results. The repeated cyclic exposure to injurious mechanical forces during tidal ventilation drives this inflammatory process further, which increases alveolar capillary barrier permeability, thereby degrading lung mechanics further and predisposing to additional VILI in a positive feedback loop.

### Biotrauma drives morbidity and mortality from VILI

Morbidity and mortality from VILI are driven primarily by the downstream effects of lung injury on other organs, that is, by biotrauma. In a classic mechanistic clinical trial from Ranieri and colleagues ^53, 58^, a lower tidal volume strategy attenuated systemic inflammation, and the difference in attenuated inflammation predicted risk of extra-pulmonary organ failures within 72 hours. In the ARDS Network tidal volume trial, lower tidal volumes similarly attenuated systemic inflammation and decreased duration of shock and renal failure, while no improvement in gas exchange over the first few days nor change in gross barotrauma was observed ^2, 59^. Therefore, protective ventilation against VILI is not only lung protective but also protective against biotrauma-mediated multiple organ failure. This multi-system effect appears to be central to its associated survival benefit.

## Monitoring for VILI at bedside

There remains no strategy for the definitive diagnosis of VILI. Molecular markers of lung injury measured in the blood that are currently used in research, such as soluble receptor for advanced glycation end-products (sRAGE) and surfactant protein D (SP-D), hold potential for use in distinguishing lung injury from cardiogenic edema and prognosticating in ARDS ^60– 62^, but they may not distinguish mechanical injury (VILI) from other forms of lung injury or ARDS more broadly. Bedside lung and respiratory system mechanics have prognostic value, but their measures do not exhibit a threshold effect for VILI—i.e. no particular numerical cutoff value differentiates VILI risk ^63– 65^—likely because of the many sources of stress and strain at play simultaneously in the injured lung. Given the association of VILI with biotrauma and multi-organ failure ^2, 53, 58, 59^, the patient with concomitant respiratory and extrapulmonary organ failures, and especially distributive shock, should be considered at high risk of VILI. Patients with certain predisposing diagnoses, including pancreatitis, post-esophagectomy, traumatic brain injury, and intracranial hemorrhage, are also classically at high risk ^8, 66, 67^. Recommendations for assessing VILI risk at bedside are reviewed in detail elsewhere ^43^.

### Monitoring tidal distension

Classic measures of VILI risk remain central to bedside management, with tidal volume and plateau pressure as surrogates of strain and stress, respectively. However, neither measure offers a data-driven threshold for risk stratification and must be interpreted in the context of a broader clinical assessment.

In ARDS, the volume of aerated lung is reduced relative to healthy lung size owing to atelectasis and edema, a phenomenon termed “baby lung” ^68, 69^. Because aerated lung volume is smaller in ARDS, smaller tidal volumes are needed ^1, 2, 53^, but there is no widely accepted strategy for individualizing tidal volume to baby lung size ^19, 64^. Airway plateau pressure, measured during an end-inspiratory pause, similarly does not account for differences in chest wall mechanics and may reflect elevations in pleural pressure, such as from obesity or a tense abdomen, for example ^22, 70, 71^.

**Figure 3.**
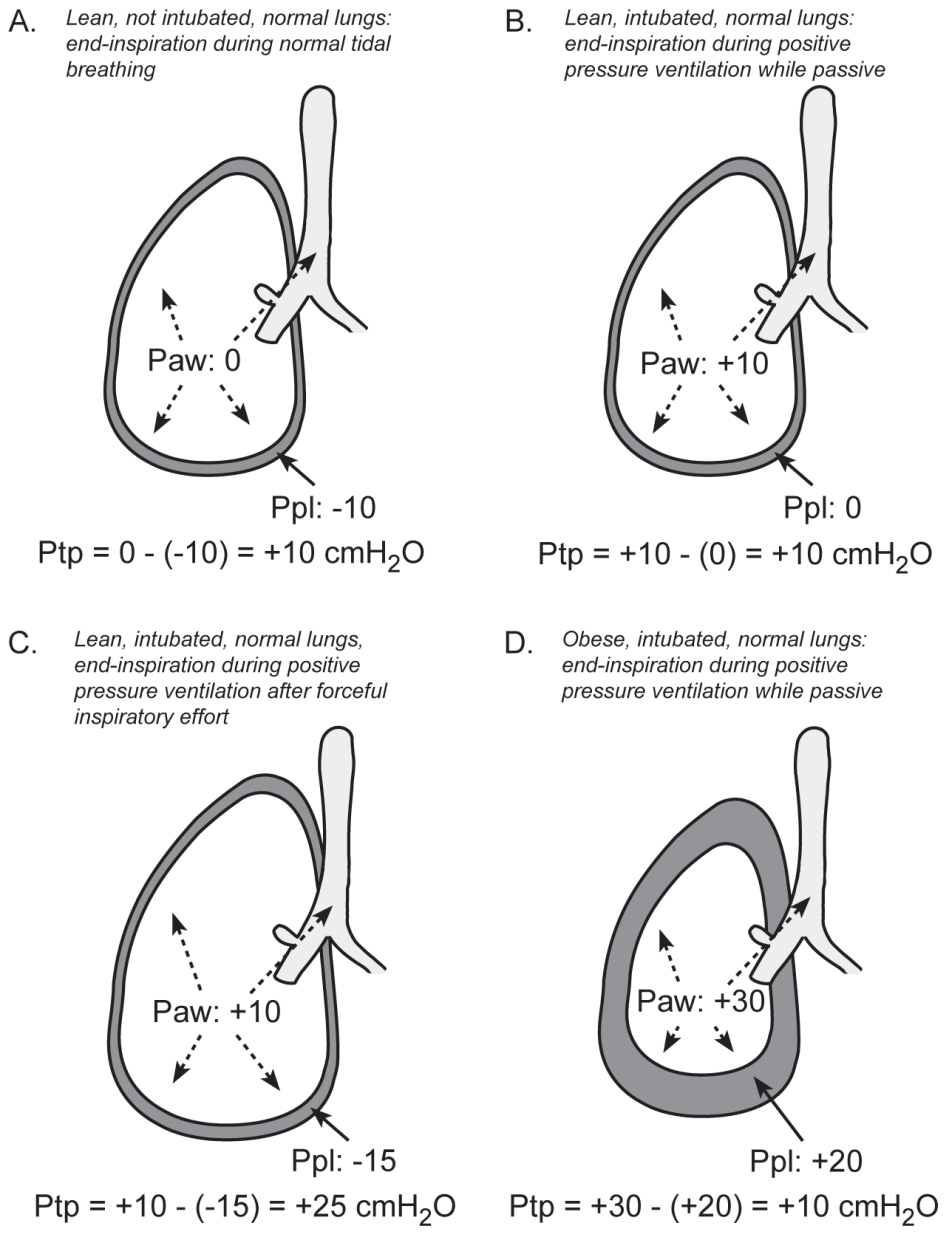
Transpulmonary pressure (Pairway – Ppleural) is the pertinent distending pressure of the lung. Measurements to ascertain lung distension at end-inspiration and end-expiration are taken during breath holds, at which time air flow is zero and airway pressure equilibrates throughout communicating airways. ( **A**) Lean, non-intubated patient with normal spontaneous tidal breathing at end inspiration. Transpulmonary pressure of 5–10 cmH _2_O is typical at end inspiration in lean healthy individuals. ( **B**) Lean, intubated patient during positive pressure ventilation at end inspiration while passive. ( **C**) Lean, intubated patient with forceful inspiratory effort has produced very high transpulmonary pressure that would be unsafe in patients at risk of ventilation-induced lung injury (VILI) despite the relatively low airway pressure. The observed transpulmonary pressure of 25 cmH _2_O is typical at total lung capacity in lean healthy individuals. ( **D**) Obese, intubated patient, with chest wall contributing high pleural pressure that results in lower transpulmonary pressure and lower lung volume at end inspiration despite higher airway pressure. Even though plateau airway pressure is relatively high, the risk of VILI is lower because lung volume and transpulmonary pressure are lower. Paw, airway pressure; Ppl, pleural pressure; Ptp, transpulmonary pressure. This figure was adapted from Beitler J, Malhotra A, and Thompson B, Ventilator-induced lung injury. *Clin Chest Med* doi: 10.1016/j.ccm.2016.07.004 with permission ^74^.

Using esophageal manometry to infer pleural pressure and calculate transpulmonary pressure (as airway minus esophageal pressure) may help overcome these limitations ( Figure 3) ^21, 72^. Esophageal pressure is measured by inserting a thin-walled balloon catheter into the mid-thoracic, retrocardiac esophagus, which positions the balloon approximately in the center of the thoracic cavity. Catheter insertion and interpretation of esophageal pressure are reviewed elsewhere ^72, 73^. Transpulmonary pressure values, a measure of global lung stress, have intrinsic meaning. For example, a transpulmonary pressure of 20–25 cmH _2_O is the typical range observed at total lung capacity in healthy individuals ^75^, and this value therefore can be thought of as the maximal stress the lungs experience in normal life. A transpulmonary pressure of 5–10 cmH _2_O is characteristic of end-inspiration during normal tidal ventilation, and a value of around 0 cmH _2_O (depending on body position) is typical in lean healthy individuals at relaxed end-exhalation ^3, 73, 76– 77^. While transpulmonary pressure is most often used for setting PEEP ^37, 78^, its potential role to guide tidal volume targets is an area of active investigation.

Airway driving pressure may be a more universally available surrogate to individualize tidal volume ^79^. Airway driving pressure, calculated as plateau pressure minus PEEP, is mathematically equivalent to the tidal volume scaled to respiratory system compliance. In an individual patient meta-analysis of multiple clinical trials, higher driving pressure was associated with increased mortality in patients with ARDS ^65^. Although there is no clear threshold effect, one might reasonably interpret values of 10 cmH _2_O or less as within the range of normal for healthy individuals based on data using esophageal manometry ^77– 79^, and thus a reasonable target range in ARDS, although heterogeneous stress distribution from alveolar interdependence may still predispose to VILI in injured lungs.

### Monitoring regional mechanics

Recent technological advances may permit monitoring regional hyperinflation and atelectrauma at bedside. Lung electrical impedance tomography (EIT) is a potentially revolutionary tool that facilitates evaluating regional lung distension in real time. Current commercial lung EIT interfaces consist of a belt, containing multiple electrodes, that is placed around the mid-chest. Images qualitatively resemble a single cross-sectional slice from a computed tomography scan (although containing different information) and are acquired non-invasively and without radiation. Technical aspects of EIT are reviewed elsewhere ^82^. EIT was used in the discovery that spontaneous effort can cause transient hyperinflation from Pendelluft ^83^, and has been used to quantify tidal opening collapse, end-tidal hyperinflation, and regional mechanical heterogeneity ^84– 86^. Current commercial devices are not without limitations ^87^. The extent to which a single cross-sectional plane from EIT can be used to make inferences about the whole lung is debatable, and EIT’s spatial resolution within that cross-sectional slice is markedly less than that of computed tomography (CT). Strategies for EIT-guided ventilation must be developed and tested in clinical trials before widespread adoption. Still, as technology and the device interface continue to improve, there is considerable potential for EIT monitoring to provide new information at bedside regarding regional ventilation that may help guide lung-protective ventilation in the future.

## Preventing VILI

### Tidal volume targets

Maintaining a tidal volume of 4–8 mL/kg PBW and a plateau pressure of ≤30 cmH _2_O are reasonable initial ventilation targets, following the ARDS Network strategy ^2^. However, recent mechanistic clinical trials suggest that lowering tidal volumes to 3–4 mL/kg PBW, using extracorporeal gas exchange as needed, further attenuates pulmonary and systemic inflammation (biotrauma) in patients with severe ARDS ^10, 11^.

Lending further weight to this possibility is the recently published, controversial EOLIA trial ^88^. EOLIA compared a conventional tidal volume strategy of 6 mL/kg PBW tidal volume with a plateau pressure of 28–30 cmH _2_O versus an “ultra-low” tidal volume strategy targeting a plateau pressure of ≤24 cmH _2_O facilitated via extracorporeal membrane oxygenation (ECMO) in patients with very severe ARDS. Sixty-day mortality was 46% with the conventional strategy versus 35% with the ECMO-facilitated ultra-low tidal volume strategy, a clinically important difference that did not achieve statistical significance. Reframing EOLIA not as an ECMO trial per se but as a VILI prevention trial means that the interpretation that ultra-low tidal volumes near 3 mL/kg PBW may confer additional benefit in very severe ARDS has sound biological plausibility, though available data are not definitive.

### Mitigating heterogeneous stress/strain distribution via prone positioning

At first glance, it might seem difficult to target heterogeneous lung insufflation with clinical management. Targeting lower tidal volume decreases the magnitude of deformation even with lung heterogeneity but does not alter the underlying heterogeneity.

Prone positioning is one strategy to facilitate more homogeneous lung aeration and more uniform strain distribution. Proning directs gravitational forces to offset forces from shape-matching of the lungs to the thoracic cavity ^89^. Indeed, this decrease in heterogeneity is thought to be a principle mechanism by which proning affords benefit ^90^. Early prone positioning was shown in a multicenter trial of moderate–severe ARDS to improve survival ^9^, and it remains underutilized in clinical practice ^91^. Discordant findings across trials of prone positioning may explain this practice inertia, but several factors explain why the latest trial may have differed from others: low tidal volumes were mandated per protocol unlike several older trials, patients were prone for at least 16 hours/day, proning was done early rather than as rescue, and proning was continued daily until patients demonstrated durable improvement in lung function ^92^.

### Atelectrauma and PEEP titration

There remains no convincing data to recommend a particular PEEP titration strategy in ARDS. Trials comparing an empiric high-PEEP strategy to a lower PEEP strategy have not demonstrated benefit with either strategy but also have not shown greater risk of barotrauma with higher PEEP ^34, 35^. In contrast, the recent Alveolar Recruitment Trial ^36^ found increased barotrauma and mortality with PEEP titrated to reduce driving pressure compared to an empiric low-PEEP strategy, but concomitant use of an unusually aggressive recruitment maneuver in the intervention arm (stepwise recruitment up to PEEP 45 cmH _2_O with driving pressure 15 cmH _2_O over several minutes) likely contributed to harm associated with the intervention.

Esophageal pressure-guided PEEP to maintain a non-negative transpulmonary pressure was compared to a low-PEEP strategy in a small single-center trial and found improved survival after adjusting for overall illness severity ^80^. However, a larger multicenter trial comparing esophageal pressure-guided PEEP to empiric high PEEP found no significant difference in mortality ^37^. Differences in the comparator arm between trials may explain these seemingly discrepant outcomes ^93^.

More broadly, uptitrating PEEP likely has tradeoffs for VILI. Any benefit of preventing atelectrauma may be offset by increasing end-tidal hyperinflation whenever end-inspiratory pressures also increase. Simultaneous lowering of tidal volume as PEEP is increased would negate exacerbating overdistension, but whether such an approach affords clinical benefit has not been tested in a clinical trial to date.

### Spontaneous breathing

The potential importance of PSILI to clinical management is yet to be defined. For mechanically ventilated patients with ARDS at greatest risk of VILI, one recent multicenter trial suggested neuromuscular blockade for 48 hours improved survival in patients with moderate–severe ARDS compared to a deep sedation strategy without neuromuscular blockade ^94^. However, a second recent trial demonstrated no discernable benefit with neuromuscular blockade when compared to a management strategy prioritizing light sedation ^95^. Several differences between the trials might explain their discordant findings ^96^, but some experts still believe there may be a role for suppressing respiratory drive in severe ARDS when forceful spontaneous respiratory efforts produce higher tidal volumes than intended ^3, 97^.

Sedation alone may not consistently eliminate patient effort ^98, 99^. Common ventilator modes, including pressure support and any form of assist-control, also are ineffective at limiting tidal volume. Even in volume-targeted modes, breath stacking dyssynchrony may yield higher volume changes than intended, generated from consecutive inspiratory cycles with incomplete exhalation between them ^97, 98, 100^.

Spontaneous respiratory effort also may play a role in regional overdistension. Spontaneous inspiratory effort during invasive ventilation can produce transient regional hyperinflation via pendelluft (transient air movement between alveoli within the lung), which theoretically also could be injurious ^83^. The extent to which this observation is clinically relevant is unknown.

Patient–ventilator interactions are often intermittent and effort variable over time. The dose response of such interactions with VILI remains unclear. Dyssynchronies such as double or reverse triggering that produce breath stacking may be injurious via repeated exposure to high tidal volumes, but intermittent exposure has unclear significance and could be beneficial to maintaining lung recruitment. As a result, the optimal management strategy also remains unclear. Whether to pursue deep sedation and/or introduce neuromuscular blockade to suppress respiratory drive and facilitate passive ventilation requires weighing several factors ^43^: biological predisposition to VILI ^61, 101, 102^, heterogeneity of regional lung mechanics ^41, 42, 103, 104^, delays in early patient mobilization with risk of ICU-acquired weakness ^105– 107^, and risk of diaphragm disuse atrophy that might prolong ventilator dependence and hospital stay ^108– 110^, among other factors. In high-risk patients with severe ARDS, frequent breath stacking in particular is likely to be injurious and warrant suppression. In cases of mild ARDS, less frequent breath stacking, or other patient–ventilator interactions, the risk/benefit of suppressing patient effort is less clear.

For the non-intubated patient with concern for PSILI, how to optimize management remains to be defined. High-flow nasal cannula exerts several physiologic effects that may be lung protective in the at-risk patient and warrant further study ^111, 112^. How best to optimize noninvasive positive pressure ventilation remains unclear and may depend on the device interface used ^113, 114^. In patients with ARDS or purely hypoxemic respiratory failure, high-flow nasal cannula appears to be the preferred noninvasive support strategy based on available evidence ^115^. Given the many co-interventions that come with invasive ventilation, prophylactic intubation seems unlikely to be beneficial, although delayed intubation in patients failing noninvasive support also may be deleterious ^116^.

## Conclusions

Over two decades after the first landmark trial from Amato and colleagues ^1^, protection against VILI remains the central treatment for ARDS. The extent to which patients without ARDS or who are not intubated experience *ventilation*-induced lung injury is unclear. Current best practice for lung protection involves weighing bedside assessment of individual patient-specific risk of VILI against tradeoffs of possible interventions. This risk/benefit analysis should be guided by expert clinical judgement with interpretation of respiratory mechanics measures that do not exhibit a threshold effect. In the years ahead, technological and molecular diagnostic advances may help guide that risk assessment in a more precise manner. While there remains no consensus on the optimal strategy to personalize ventilatory support for VILI protection, there is universal agreement that protection against VILI improves morbidity and mortality in at-risk patients.

## Abbreviations

ARDS, acute respiratory distress syndrome; ECMO, extracorporeal membrane oxygenation; EIT, electrical impedance tomography; PEEP, positive end-expiratory pressure; PBW, predicted body weight; VILI, ventilation-induced lung injury.
